# Protein arginine methyltransferase 1 in the generation of immune megakaryocytes: A perspective review

**DOI:** 10.1016/j.jbc.2022.102517

**Published:** 2022-09-21

**Authors:** Xinyang Zhao, Zechen Chong, Yabing Chen, X. Long Zheng, Qian-Fei Wang, Yueying Li

**Affiliations:** 1 Department of Biochemistry and Molecular Genetics, The University of Alabama at Birmingham, Birmingham, Alabama, USA; 2 Department of Genetics, The University of Alabama at Birmingham, Birmingham, Alabama, USA; 3 Department of Pathology, The University of Alabama at Birmingham, Birmingham, Alabama, USA; 4 Department of Pathology and Laboratory Medicine, University of Kansas Medical Center, Kansas City, Kansas, USA; 5 Chinese Academy of Sciences (CAS) Key Laboratory of Genomic and Precision Medicine, Beijing Institute of Genomics, Chinese Academy of Sciences and China National Center for Bioinformation, Beijing, China

**Keywords:** protein arginine methyltransferase 1, PRMT1, phosphatase, RNA-binding protein, RNA splicing, megakaryocyte, platelet, inflammation, innate and adaptive immunity, thrombosis, HSC, hematopoietic stem cell, LPS, lipopolysaccharide, MDS, myelodysplasia syndrome, Mks, megakaryocytes, PRMT, protein arginine methyltransferase, scRNA-seq, Single-cell RNA sequencing, vWF, von Willebrand factor

## Abstract

Megakaryocytes (Mks) in bone marrow are heterogeneous in terms of polyploidy. They not only produce platelets but also support the self-renewal of hematopoietic stem cells and regulate immune responses. Yet, how the diverse functions are generated from the heterogeneous Mks is not clear at the molecular level. Advances in single-cell RNA seq analysis from several studies have revealed that bone marrow Mks are heterogeneous and can be clustered into 3 to 4 subpopulations: a subgroup that is adjacent to the hematopoietic stem cells, a subgroup expressing genes for platelet biogenesis, and a subgroup expressing immune-responsive genes, the so-called immune Mks that exist in both humans and mice. Immune Mks are predominantly in the low-polyploid (≤8 N nuclei) fraction and also exist in the lung. Protein arginine methyltransferase 1 (PRMT1) expression is positively correlated with the expression of genes involved in immune response pathways and is highly expressed in immune Mks. In addition, we reported that PRMT1 promotes the generation of low-polyploid Mks. From this perspective, we highlighted the data suggesting that PRMT1 is essential for the generation of immune Mks *via* its substrates RUNX1, RBM15, and DUSP4 that we reported previously. Thus, we suggest that protein arginine methylation may play a critical role in the generation of proinflammatory platelet progeny from immune Mks, which may affect many immune, thrombotic, and inflammatory disorders.

Protein arginine methylation is a common protein modification that only occurs in eukaryotic cells. A family of protein arginine methyltransferases (PRMTs) has at least nine members, which either symmetrically or asymmetrically modify the guanidino nitrogen atoms of the arginine side chain in the context of proteins. The basic molecular functions of these enzymes were comprehensively reviewed (1, 2). Among the family members, PRMT1 is the most evolutionarily conserved enzyme existing from yeast to mammals and accounts for most of the asymmetric arginine methylation activity. PRMT1 participates in a broad range of molecular processes such as transcriptional regulation, RNA splicing, protein trafficking, protein translation, and signal transduction (3). PRMT1 is ubiquitously expressed in all tissues with the highest expression levels observed in the female reproductive system (https://www.gtexportal.org/home/). Straight knockout of PRMT1 is embryonic lethal in mice. PRMT1 has been demonstrated to be critical for cardiovascular fitness (4, 5, 6, 7), pancreatic development (8), and brain development (9, 10). High expression of PRMT1 has been linked to poor overall survival in many types of cancers; conversely, inhibition of PRMT1 activity blocks the proliferation of leukemia and solid tumors such as lung, breast, and ovarian cancers.

Arginine methylation like phosphorylation is involved in the relay of extracellular and intracellular signals. PRMT1 activity and subcellular localization are regulated by phosphorylation. The yeast homolog of PRMT1 (Hmt1) has been shown to be inactivated through dephosphorylation in response to starvation, although the corresponding phosphorylation site on Hmt1 is not available in mammalian PRMT1 (11). The casein kinase 1A1 gene (*CSNK1A1*) is located within the del(5q) in myelodysplasia syndrome (MDS). Haploinsufficiency of *CSNK1A1* in *Csnk1a1*^*−/+*^ KO mice causes mild dysplasia of megakaryocytes (Mks) in the bone marrow (12). Casein kinase 1A phosphorylates PRMT1 and directs it to chromatin (13). Thus, haploinsufficiency of *CSNK1A1* may affect PRMT1-mediated epigenetic regulation in megakaryopoiesis. There are many potential phosphorylation sites on PRMT1 (14, 15), although systematic studies on how phosphorylation changes PRMT1 activity have not been performed. In turn, PRMT1 methylates and activates various kinases. In leukemia cells, PRMT1 methylates and activates FLT3 kinase and FLT3 ITD kinase, which are commonly found in acute myelogenous leukemia (16). Furthermore, epidermal growth factor receptor is methylated by PRMT1 in triple-negative breast cancer cells, and methylation enhances epidermal growth factor receptor activity (17). The expression of PRMT1 is upregulated by insulin and cytokines in cell lines (18, 19), whereas the expression of PRMT1 is downregulated by nutritional stress (20). PRMT1 is required for the activation of SMAD signaling *via* methylation of the suppressor SMAD6 (21) and for activation of the mTOR pathway (20). PRMT1 also interacts with the interferon alpha receptor and promotes the interferon response (22). Further still, the Wnt signaling pathway is activated by PRMT1 through the methylation of GSK3 kinase (23). Taken together, the variation in PRMT1 activity further diversifies the outcomes of phosphorylation-mediated signaling and thus contributes to cell heterogeneity.

PRMT1 expression levels are varied in blood cells with the lowest in hematopoietic stem cells (HSCs) and the highest in megakaryocyte-erythroid progenitors (24). *PRMT1* knockout in *Mx1*-cre mice increased the number and polyploidy of Mks in the bone marrow but caused bone marrow failure a few weeks after the induction thereof (25). Here, we summarize our findings on how PRMT1 regulates megakaryopoiesis at the molecular level and offer our perspective on the novel roles of PRMT1 in the generation and the functions of a group of immune Mks.

## Multiple routes for the generation of Mks

**Figure 1:**
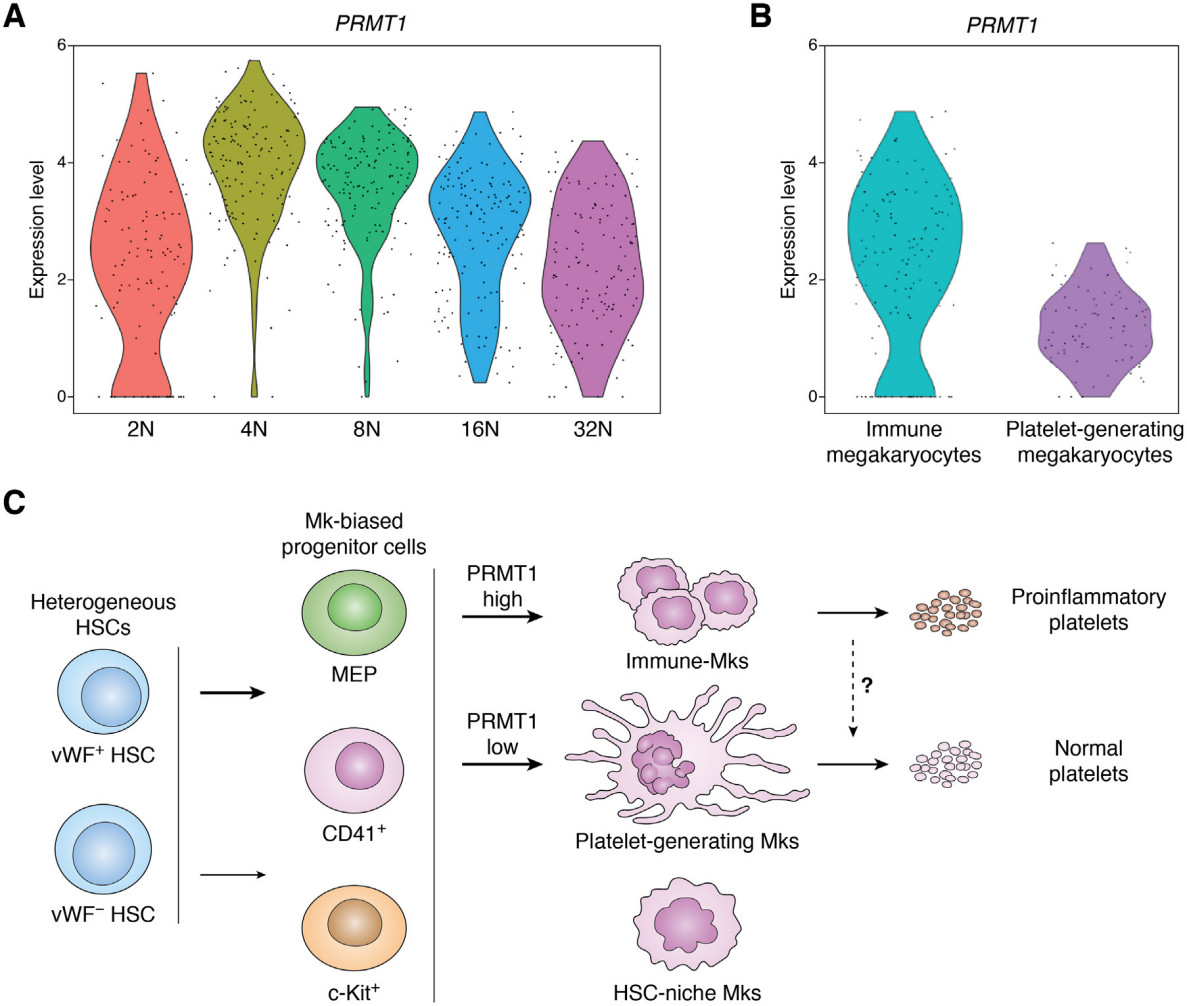
***PRMT1* expression is higher in low-polyploid immune Mks.** Univariate analysis of scRNA data (46). *A*, compare *PRMT1* expression in human Mks with different polyploidy. *B*, *PRMT1* expression levels in immune Mks (CD53^+^CD41^+^ or LSP1^+^CD41^+^) *versus* platelet generation Mks. *C*, potential role of PRMT1 in megakaryopoiesis. HSCs are heterogeneous. A group of HSCs expressing vWF is biased toward megakaryocyte differentiation, although they can differentiate into other blood lineages. Several types of progenitor cells are biased toward megakaryocyte differentiation. These progenitor cells (SL-MkPs) express CD41 on the cell surface, along with markers for phenotypic HSCs. Common myeloid progenitor cells expressing CD41 are unipotent for megakaryocyte differentiation. In addition, MEPs retain the binary differentiation capability for both erythroid and megakaryocyte differentiation. MEP cells expressed the highest levels of PRMT1. When progenitors undergo further differentiation into mature megakaryocytes, the activity of PRMT1 determines the direction to immune Mks or platelet-generating Mks and HSC-niche Mks. HSC, hematopoietic stem cell; MEP, megakaryocyte-erythroid progenitor; Mks, megakaryocytes; PRMT, protein arginine methyltransferase; SL-MkP, stem-like megakaryocyte progenitors; vWF, von Willebrand factor.

Single-cell RNA sequencing (scRNA-seq) technology elucidates the heterogeneity and continuum within HSCs and hematopoietic progenitors of all blood lineages (26, 27, 28, 29, 30, 31, 32). HSCs give rise to megakaryocyte-erythroid progenitor cells, which further differentiate into Mks in the bone marrow under normal homeostatic conditions (33, 34). Single-cell sequencing analysis suggests that Mks may be generated from more than one type of progenitor or stem cell (Fig. 1) (35). Both platelets and Mks store the von Willebrand factor (vWF) protein, a complex plasma glycoprotein that modulates platelet adhesion at the site of a vascular injury. A subpopulation of HSCs expressing high levels of vWF protein tends to differentiate into Mks with high efficiency even though these vWF^+^ HSCs retain their capability to generate other blood lineages in bone marrow transplantation assays (36). Phenotypic HSCs expressing high levels of c-Kit (a tyrosine kinase receptor for stem cell factor) differentiate more efficiently into Mks but lose their self-renewal capability (37). Thrombopoietin receptor (also known as *MPL* or *c-MPL*) is required for Mk production. In *c-Mpl*^*−/−*^ mice, the frequency of a group of multipotent progenitors, namely MPP2, expressing CD41 was reduced, whereas the other multipotent progenitor populations did not change. Subsequently, MPP2 was exclusively responsible for the generation of Mks (38). Consistently, CD41^+^CMP (common myeloid progenitors) have been shown to generate only Mks (39). The CD41^+^CD38^−^CD34^+^ progenitors are committed for Mk differentiation (33). Challenged with lipopolysaccharide (LPS), quiescent but primed stem-like megakaryocyte progenitors, which express high levels of CD41 within the phenotypic long-term HSCs, quickly expand and undergo maturation and protein synthesis to generate platelets (40). Altogether, these studies suggest that although CD41 is a common marker for Mk cells at different developmental stages, there are many paths or many subgroups of HSCs or hematopoietic progenitors that can generate mature Mks, which may account for Mk heterogeneity and hence platelet heterogeneity.

## A subgroup of Mks as uncharacterized immune cells

Evidence of Mks as immune cells existed before scRNA-seq technology was applied (41). Transplantation of Mks expressing interleukin-1 can induce arthritis (42). Mks activate T cells such as Th17 *via* major histocompatibility complex (MHC) class II protein expressed on the Mk surface (42). Recently, scRNA-seq technology has demonstrated that heterogeneity also exists in mature blood cells such as neutrophils (43) and Mks in both humans and mice (35, 44, 45, 46, 47, 48). Mks residing in the bone marrow have multiple nuclei, which account for their large sizes ranging from 50 to 100 μm. In addition to their sizes, their low number in the bone marrow makes handling fragile Mks challenging in experiments. Therefore, characterization of Mks especially the large polyploid Mks is often missed in regular scRNA-seq analysis. Sun *et al.* (46) utilized a large nozzle and a lower flow rate to isolate Mks based on surface markers, namely CD41, and polyploid status and then applied the scRNA-seq method (Smart-seq2) to collect the transcriptome profiles of individual Mks. For smart-seq2, Mks spanning all degrees of ploidy were collected. In contrast, the automated cell collection procedure employed by 10X Genomics preferentially enriches for low-ploid cells, while high-ploid Mks have limited access to channels of 10X microfluid and may not be sorted because of their fragility and large size (>65 μm in diameter). Moreover, Smart-seq2 detected more genes in a cell, especially low-abundance transcripts, as well as alternatively spliced transcripts than did 10X Genomics Chromium (49). Mks were clustered into several groups: platelet-generating Mks, immune Mks, and HSC-niche Mks in addition to a group of Mks that expressed high levels of cell cycle genes. Both mice and humans have the immune-Mk subgroups that express immune response genes and transcription factors; PU.1 that is known to promote monocyte differentiation and suppress late megakaryopoiesis (50) and IRF8 that activates proinflammatory gene expression (26). Therefore, immune Mks may resemble partial functions of monocytes but do not express CD11b, as do typical monocytes. Consistent with the immune phenotype, immune Mks also express high levels of Runt-related transcription factor 1 (*RUNX1*) (51), *CIITA* (51), *STAT1* (52), and *NFKBBIZ* (52), which regulate immunity. In addition to transcription factors, immune response genes such as *HLA-DQA1*, *LILRB1*, *CSF2RA*, *CSF3R*, and *IL1R2* are differentially and highly expressed in immune Mks. In contrast, platelet-generating Mks express high levels of *GATA2*, *NF-E2*, and *FLI-1*, transcription factors that are well known for inducing Mk maturation.

After analyzing the differentially expressed genes between different subgroups, Sun *et al.* utilized the cell membrane proteins CD53 and CD41 to sort the immune Mks for *in vitro* functional assays. They demonstrated that the CD53^+^CD41^+^ population expanded in response to LPS challenge within the first 12 h with an upregulation of a preexisting PU.1 (encoded by the *SPI1* gene) and IRF8-associated inflammatory transcription. Isolated primary immune Mks were capable of engulfing and ingesting bacterial particles, as well as activating T cell expansion when cocultured with T cells *in vitro* in the presence of anti-CD3 and CD28 antibodies. These murine immune Mks express higher levels of MHC class II molecules when stimulated with LPS, implying that immune Mks can present foreign antigens during infection.

The existence of immune Mks in human adult bone marrow was independently verified by Liu *et al.* (44) using Smart-seq2. They identified two surface markers, CD48 and CD148, that define immune Mks. When mice were challenged with LPS or interferon gamma, the CD48^+^CD148^+^ Mk population expanded in number and expressed high levels of Toll-like receptor 4 (*TLR4*), *TLR2*, S100 calcium-binding protein A8 (*S100A8A*), and other proteins related to inflammatory response. The existence of immune Mks has also been demonstrated by Pariser *et al.* (45) using Mks isolated from the lungs. Strikingly, lung Mks express CD53, LSP1, CD48, and CD148 on the surface, similar to immune Mks in the bone marrow (44, 46). Whether immune Mks coexpress CD53 and CD48 on the same cells has not yet been investigated using flow cytometry. Unlike bone marrow–derived Mks, lung Mks are generally smaller in size (53). Lung Mks express MHC class II complexes and activate T cells when primed with ovalbumin (OVA) protein, which is widely used as an antigen for presentation. In addition to antigen presentation, lung Mks express genes involved in inflammatory reactions (48, 53). Therefore, adult lung Mks are functionally similar to immune Mks from the bone marrow. Further proof is provided in the same Sun *et al.* study, in which scRNA analysis of lung-derived Mks and immune Mks from bone marrow were compared. Both groups of Mks had enriched immune-related gene expression signatures. The two types of immune Mks have comparable levels of the characteristic markers, LSP1 and CD53, and transcription factors SPI1/PU.1 and IRF8. By comparing bone marrow–derived Mks with lung-derived Mks from published adult and fetal scRNA data (45, 48), Sun *et al.* revealed that immune Mks are conserved and exist in different tissues (bone marrow or lung) and developmental stages (fetal or adult). While adult lung Mks were enriched with transcriptional signatures of immune-related processes, including pathogen recognition, phagocytosis, and antigen presentation, fetal lung and adult bone marrow immune Mks showed differential expression signatures in phagocytosis and antigen presentation, respectively (Table 1). These differences might be explained by the fact that lung-derived Mks are constantly exposed to bacteria, viruses, and other environmental hazards. An alternative explanation is that 5% of Mks have neutrophils inside their cells *via* emperipolesis, which is stimulated by LPS (41). Thus, it is possible that the inflammatory signature may partially reflect the genes expressed in neutrophils inside some of the adult lung-derived immune Mks.

**Table 1:** Differentially expressed genes in immune Mks from different tissues (46)

Tissue	Genes and functions
Adult bone marrow	Platelet functions: *Pf4*, *Flna*, *Nfe2*, *Serpine 2*, *Vwf*, *Ppbp* Antigen presentation: *Cd74*, *H2-Ab1*, *H2-Eb1*, *H2-DMa*, *H2-DMb1* Anti-bacteria peptides: *S100a8*, *S100a9*, *Camp*
Adult lung	Antigen presentation: *Cd74*, *H2-Ab1*, *H2-Eb1*, *H2-DMa*, *H2-DMb1* Phagocytosis: *C1qa*, *C1qb*, *C1qc*, *C5ar1*, *Ctsh*, *Ctsc*, *Ctsb*, *Ctsz*, *Ctsd*, *Ctsl*, *Lamp1*, *Fcgr3* Chemotaxis: *Cxcl16*, *Cd14*, *Cxcl2*, *Ccl2*, *Ccrl2*
Fetal lung	Phagocytosis: *C1qa*, *C1qb*, *C1qc*, *C5ar1*, *Ctsh*, *Ctsc*, *Ctsb*, *Ctsz*, *Ctsd*, *Ctsl*, *Lamp1*, *Fcgr3*

Another scRNA-seq analysis of Mks further confirmed a subgroup of Mks expressing genes with an immune response signature, including *CD53*, as described in other groups (54). They used CXCR4 as an additional marker to isolate immune Mks for additional immunological assays. They discovered that CXCR4-high Mks migrated to the spleen and liver upon stimulation by bacterial infection. *CXCR4*^*high*^ Mks secrete IL-6 and TNFα and contact myeloid cells as an adaptive immune response in addition to an enhanced innate immune response. In an early study, low-polyploid Mks were shown to express MHC class II molecules, whereas high-polyploid Mks expressed MHC class I (55). Interestingly, since *CXCR4*^*high*^ Mks express MHC class II molecules, we suspect that these Mks are not fully mature or able to generate platelets. To date, platelets expressing MHC class II molecules have not been reported.

Whether CD53^+^CD41^+^ Mks can recapitulate all the biological functions of immune Mks, as defined by scRNA-seq analysis, requires further investigation. Both MHC classes I and II are involved in antigen presentation in Mks (42, 56). MHC class I is expressed in mature Mks (42). Accordingly, MHC class I gene expression was observed in all four groups defined in our scRNA-seq studies. Since none of the MHC markers or PRMT1 were exclusively expressed in a specific group, a low percentage of high-polyploid Mks may still express PRMT1 and participate in immune reactions.

## Immune Mks may be responsible for generating proinflammatory platelets

Both high- and low-polyploid Mks can produce platelets, although high polyploidy may do so more efficiently (57). Therefore, the heterogeneity of Mks determines the heterogeneity of platelets. Neonatal platelets are less active in response to stimulants than adult platelets (58), which may be due to the low polyploidy of fetal Mks (59). Immune Mks were enriched in the fraction of low-polyploid Mks (46). Lung Mks, like bone marrow immune Mks, have low polyploidy (48). Platelet budding from lung Mks has been visualized *in vivo* using two-photon microscopy (53). Given the transcriptome similarity between lung Mks and bone marrow immune Mks, it is possible that immune Mks in bone marrow like lung Mks (53) can produce platelets. Although immune Mks express genes responsible for inflammation, whether platelets generated from immune Mks are proinflammatory has not been tested experimentally. According to scRNA-seq analysis (47), human fetal liver and embryo yolk sac cells contain Mks. Further analysis demonstrated that a subpopulation of early embryonic Mks expresses immune response genes (47), suggesting that there may be two developmental paths of megakaryopoiesis, with one directing toward Mks expressing immune-responsive genes and the other toward platelet production. However, according to the Mk distribution along these developmental paths, it cannot be ruled out that immune Mks are in transit to become platelet-producing Mks. Inflammation can be a prerequisite for platelet production in certain progenitors. Intriguingly, these stem-like megakaryocyte progenitors also express interferon response genes that are critical for platelet generation, as *IFNAR*^−/−^ mice cannot produce Mk-specific proteins in these phenotypic HSCs (40). These data are consistent with the observation that STAT1 activation in interferon signaling promotes megakaryopoiesis (60).

Many clinical studies have shown that platelets play a role not only in thrombus formation but also in innate immunity, inflammation, and carcinogenesis (61, 62, 63, 64). Platelets from patients with sepsis and COVID-19 have different transcriptome profiles and protein components compared to those from normal human controls (62, 63, 64). Under septic conditions, immune Mks may expand and produce high levels of pathogenic platelets that express high levels of inflammatory response genes. Therefore, determining the molecular mechanism underlying the differentiation of immune Mks is critical for uncovering the genesis of proinflammatory platelets.

## Protein arginine methylation for the generation of immune Mks

Based on our published data on PRMT1-mediated megakaryopoiesis and its direct effects on the protein concentrations of the transcription factor PU.1, the RNA-binding motif protein 15, RBM15, and dual specificity phosphatase 4 (DUSP4), we argue that protein arginine methylation plays a critical role in the generation of immune Mks. Our scRNA-seq analysis of human cord blood cells stimulated with thrombopoietin and stem cell factor for MK differentiation revealed that the expression of PRMT1 was positively correlated with the expression of proinflammatory genes in individual Mks (65). We previously demonstrated that PRMT1 expression plays a critical role in megakaryocyte differentiation (24, 66). Constitutively high expression of PRMT1 blocks Mks from further developing into high-polyploid cells. In addition, our unpublished data from mice conditionally overexpressing PRMT1 under the platelet factor 4 (PF4) promoter, which drives the expression of PRMT1 mainly in Mk-committed cells, demonstrated that, in 6-week-old mice, overexpression of PRMT1 leads to decreased polyploidization with no effect on platelet count. Conversely, inhibition of asymmetric methyltransferase activity with the potent inhibitor, MS023 at 50 mg/kg, increases platelet count and polyploidy in mice (65). Given that the majority of immune Mks have low polyploidy (≤8N), we further investigated PRMT1 expression levels using the scRNA-seq data reported by Sun *et al.* (46). We found that PRMT1 was highly expressed in immune Mks and in the low polyploidy fraction (Fig. 1), which is consistent with our findings that high expression of PRMT1 blocks Mk differentiation into high-polyploid cells. PRMT1 is largely expressed in the bone marrow blood cells of patients with MDS. Given that thrombocytopenia is an ominous symptom of MDS, our findings support the use of PRMT1 inhibition to combat this symptom in affected patients (65).

**Figure 2:**
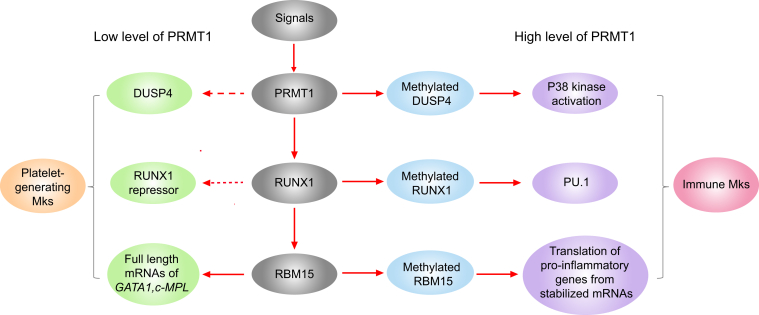
**The generation of immune Mks is dependent of PRMT1 dose according to our perspectives.** Many signals have been shown to activate PRMT1 *via* increasing its protein levels or phosphorylation. According to our studies, once PRMT1 is upregulated, PRMT1 can methylate DUSP4, RUNX1, and RBM15. Methylation of DUSP4 and RBM15 leads to their degradation; thus, p38 kinase is maintained in activation status, and the mRNAs of proinflammatory genes are stable because of RBM15 degradation. PRMT1 upregulation also converts RUNX1 into a transcriptional activator of PU.1 *via* methylation. PU.1 activates the transcription of innate immune response genes. Collectively, these results indicate that PRMT1 upregulates the generation of immune Mks. In contrast, PRMT1 downregulation upregulates DUSP4 and RBM15 and converts RUNX1 to a transcriptional repressor. RBM15 is required for the generation of full-length GATA1 and c-Mpl mRNAs *via* RNA splicing. Given that RBM15 transcription is dependent on RUNX1 activation, transient upregulation of PRMT1 may be required for the production of RBM15. Downregulation of PRMT1 is required for the generation of platelet-generating Mks. Thus, the dynamic regulation of PRMT1 is required for optimal differentiation. Mks, megakaryocytes; PRMT, protein arginine methyltransferase.

Genes regulated by RUNX1, RBM15, and DUSP4 in the presence of different levels of PRMT1 expression may determine the direction of Mk differentiation into immune or platelet-generating Mks (Fig. 2). Mechanistically, we have identified that methylation of RUNX1 by PRMT1 disrupts its binding to a transcriptional repressor complex, the mSIN3 complex, thus activating PU.1 transcription (66). PU.1 is a master transcription factor that drives monocyte-granulocyte differentiation and blocks Mk differentiation (50). Consistently, we discovered that PU.1 is repressed by RUNX1 when PRMT1 is downregulated during Mk differentiation. RUNX1 is also required for the maturation of Mks and polyploidization, as a transcriptional repressor of the *MYH10* gene, which encodes nonmuscle myosin IIB heavy chain protein for blocking polyploidization (67).

The gene encoding RBM15 is part of a recurrent chromosome translocation, t(1;22), in acute megakaryoblastic leukemia (68, 69). Downregulation of RBM15 promotes the generation of Mk progenitors, while RBM15 is required for Mk maturation into high-polyploid cells (70). Furthermore, methylation of RBM15 by PRMT1 triggers its degradation *via* CNOT4-mediated ubiquitination, whereas overexpression of RBM15 can rescue the PRMT1-mediated block of Mk differentiation (24). RBM15 may control the stability of mRNAs *via* binding to the 3′UTR; thus, methylation of RBM15 by PRMT1 stabilizes the mRNAs involved in inflammatory responses, such as *IL-16*, *IL12RB2*, *IL1A*, and *IL18BP*. Additionally, RBM15 controls RNA splicing to produce full-length mRNAs of many genes, including *GATA1*, *RUNX1*, *TAL1*, and *c-MPL*, which are required for Mk maturation and polyploidization (24, 70, 71, 72). Given that PRMT1 promotes the alternative splicing of *c-MPL* RNAs that encode for truncated c-MPL proteins, which respond poorly to thrombopoietin, Mks expressing high levels of PRMT1 may be less efficient in producing platelets. We previously reported that RUNX1 activates the transcription of *RBM15* (73). Paradoxically, RBM15 protein is degraded by PRMT1-mediated ubiquitylation. As a result, along with the activation of PU.1, Mks expressing PRMT1 remain immune Mks. Because RBM15 is required for MK polyploidization, downregulation of PRMT1 is necessary for extending the stability of RBM15 protein. Nevertheless, activation of PRMT1 is still required for the initiation of megakaryopoiesis *via* RUNX1-mediated transcriptional activation of RBM15. Thus, the timing and amplitude of transcriptional activation by PRMT1 and RUNX1 determine the protein levels of RBM15, which may further determine the heterogeneity of mature Mks (Fig. 2). Furthermore, Myc is required for generation of Mk progenitors, whereas its downregulation is required for platelet generation (74). Since PRMT1 has a consensus E-box, Myc may activate PRMT1 during megakaryopoiesis.

Recently, we discovered that DUSP4 is a critical downstream target of PRMT1 in megakaryopoiesis (65). Methylation of DUSP4 by PRMT1 triggers methylation-dependent recruitment of the E3 ligase, HUWE1, which triggers the degradation of methylated DUSP4. Ectopic expression of DUSP4 rescues PRMT1-mediated blockage and promotes polyploidization of mature Mks. Consistently, knockdown of HUWE1 promotes MK differentiation. We found that DUSP4 specifically dephosphorylates p38 mitogen-activated protein (MAP) kinase in Mks. Activation of MAPK1, a MAP kinase, is required for megakaryopoiesis (75), whereas activation of p38 MAP kinase blocks megakaryopoiesis (76). Given that p38 MAP kinase is often activated in inflammatory reactions (77), it is likely that PRMT1-regulated DUSP4 degradation indirectly activates the p38 MAP kinase pathway to promote inflammation. Consistently, the activation of p38 kinase has been revealed in Mks isolated from primary fibrosis (76) and MDS (65, 78), which leads to defective megakaryopoiesis. Observations in DUSP4 KO mice have demonstrated the critical role of DUSP4 in the inflammatory response to infection (79, 80). DUSP4 is a nuclear phosphatase which binds to other substrates, such as chromatin-bound proteins (81). Accordingly, PRMT1 can also participate in a p38 kinase–independent pathway, *via* methylation of DUSP4. For example, DUSP4 may affect epigenetic regulation by modulating phosphorylation of chromatin-bound proteins such as phosphorylated H3S10 (82). This requires further investigation.

## Perspectives

We have developed a novel vital fluorescent dye to label intracellular PRMT1 so that we can isolate live cells according to PRMT1 expression levels for functional studies (83). How immune Mks expressing high levels of PRMT1 may be expanded under disease conditions *in vivo* requires further investigation. Targeting PRMT1 has been shown to generate neoantigens in cancer cells, accounting for its high efficacy in cancer treatment in combination with immune checkpoint blockers (84, 85). Platelets are known to promote cancer proliferation and metastasis (86), and whether platelets generated in cancer patients are different from those generated in healthy individuals has been explored (87). However, whether platelets from cancer patients are generated by immune Mks is not yet known. Here, we argue that targeting PRMT1, which blocks the generation of immune Mks and their pathogenic platelet progenies, may create a hostile tumor microenvironment for cancer cells to adapt to. Recently, inhibition of PRMT1 in combination with PD-1 immune checkpoint blockers has shown promising results in the treatment of melanoma (88). Understanding PRMT1’s role in generating immune Mks will help to elucidate the molecular mechanisms underlying immune therapy.

Platelets carry granules that contain pre-mRNAs from Mks (89). Upregulation of PRMT1 in immune Mks activates RUNX1-mediated transcription, RBM15-mediated RNA processing, and DUSP4-controlled signaling events, which could induce Mks to produce platelets with already spliced mRNAs encoding for proinflammatory cytokines and changes in surface proteins that alter the communication between platelets and immune cells, such as monocytes, macrophages, and T cells (90). Platelets from patients with severe COVID-19 have activated p38 kinase (91). Mks have been detected from pulmonary and cardiac systems including brain capillaries in COVID-19 patients (92). We speculated that immune Mks migrate to lung and cardiac tissues in large amount especially in severe COVID-19 patients and that COVID-19 platelets may be generated from immune Mks, expressing higher levels of PRMT1 in the lungs, which lead to severe thrombosis. The number of interferon-activated Mks increases in severe COVID-19 cases according to multiomics analysis (93). The contribution of Mks to cytokine storms was implied in another scRNA analysis of COVID-19 immune cells (94). Thus, it is plausible to predict that targeting PRMT1 could be used to alleviate thrombo-inflammation in patients with severe COVID-19. In addition, platelets are etiological factors for cardiovascular diseases, including atherosclerosis (95). Pathogenic platelets from PRMT1-controlled immune Mks may establish and accelerate atherosclerosis progression. Apart from platelets, immune Mks may communicate independently with tumor cells and other immune cells during pathogenesis.

Taken together, we posit that PRMT1 plays a critical role in the generation of immune Mks. Intensive research is urgently needed to study the genesis of both immune Mks and their pathogenic platelet progenies and to determine their roles in innate and adaptive immune responses to infection, cancers, and cardiovascular diseases.

## Dataset

Sun, S., Jin, C., Si, J., Lei, Y., Chen, K., Cui, Y., Liu, Z., Liu, J., Zhao, M., Zhang, X., Tang, F., Rondina, M. T., Li, Y., and Wang, Q. F. (2021) Single-cell analysis of ploidy and the transcriptome reveals functional and spatial divergency in murine megakaryopoiesis. Genome Sequence Archive for Human, HRA000114.

## Conflict of interest

X. L. Z. is a consultant for Alexion, Takeda, Sanofi-Genzyme, and BioMedica. X. L. Z. is also a cofounder of Clotsolution. All other authors declare no conflict of interest.
